# Renal hydatid cyst mimicking malignancy: a case report

**DOI:** 10.1016/j.ijscr.2025.111506

**Published:** 2025-06-13

**Authors:** Maryam Maghbool, Mohammad Reza Dehghani, Babak Samizadeh

**Affiliations:** a Clinical Research Development Unit of Valiasr Hospital, Fasa University of Medical Sciences, Fasa, Iran; b Student Research Committee, Fasa University of Medical Sciences, Fasa, Iran

**Keywords:** Echinococcosis, Hydatid cyst, Renal, Kidney, Cyst, Nephrectomy

## Abstract

**Introduction and importance:**

Hydatid disease, caused by Echinococcus granulosus, primarily affects the liver and lungs, with renal involvement being rare. Renal hydatid cysts are often misdiagnosed as malignancies due to their cystic nature and similar imaging characteristics. Misdiagnosis can lead to inappropriate treatment, making it crucial to include hydatid disease in the differential diagnosis of renal cystic lesions, especially in endemic regions. Early detection, accurate diagnosis, and timely management are essential for preventing complications and achieving favorable outcomes.

**Case presentation:**

We present the case of a 59-year-old woman with right flank pain and gross hematuria. Imaging, including ultrasound and a contrast-enhanced CT scan, revealed a large cystic lesion with calcifications in the right kidney, leading to a preliminary diagnosis of malignancy. The patient underwent laparotomic nephrectomy without preoperative medical therapy. Postoperative histopathological examination confirmed the presence of a renal hydatid cyst. Following surgery, the patient was placed on a three-month course of albendazole to prevent recurrence. Six months later, she remained asymptomatic with normal renal function and no evidence of hydatid cysts elsewhere.

The patient did not receive preoperative albendazole therapy, which is commonly recommended to prevent cyst dissemination during surgery. In this case, the decision to proceed without preoperative medical therapy was due to the mistaken diagnosis of malignancy. This underscores the importance of considering hydatid cysts in the differential diagnosis before deciding on a surgical approach.

The decision to perform a radical nephrectomy was driven by the presumption of renal malignancy. Alternative surgical options, such as cystectomy or conservative surgery, were not considered due to the initial misdiagnosis. Had hydatid disease been suspected, a less aggressive approach aimed at cyst removal and kidney preservation could have been attempted.

**Discussion:**

Renal hydatid cysts are rare and challenging to diagnose due to their resemblance to renal tumors. Surgical resection is the preferred treatment, especially when malignancy is suspected. This case highlights the importance of postoperative medical therapy to prevent recurrence, as well as the need for histological confirmation to guide appropriate management.

Long-term follow-up of 12–24 months is recommended to monitor for recurrence of hydatid disease. In this case, follow-up was limited to six months, which may not be sufficient to entirely exclude the possibility of recurrence.

**Conclusion:**

This case underscores the importance of considering hydatid disease in renal cystic masses, emphasizing early diagnosis, surgical management, and postoperative prophylaxis for optimal outcomes.

## Highlights

• Renal hydatid cysts are rare and can be misdiagnosed as malignancies due to overlapping radiologic features.
• Early detection and differentiation between hydatid cysts and renal tumors are critical for appropriate treatment.
• Surgical intervention, such as nephrectomy, is often necessary when malignancy is suspected.
• Postoperative prophylaxis with albendazole is essential to prevent recurrence and ensure favorable long-term outcomes.

## 1 Introduction

Renal hydatid cyst, a less common form of hydatid disease, is caused by the larval stage of the parasitic tapeworm Echinococcus granulosus. While this disease primarily affects the liver and lungs, it can also impact other organs, including the kidneys, which account for only 2–4 % of cases. Renal hydatid cysts often remain asymptomatic for an extended period, going undiagnosed until the cyst grows large enough to cause clinical symptoms. These symptoms may include flank pain, hematuria, or a palpable mass. Diagnosis is typically confirmed through imaging techniques such as ultrasonography (US) or computed tomography (CT), supported by serological tests [1,2].

Treatment options for renal hydatid cysts involve a combination of pharmacotherapy with albendazole and surgical interventions. The choice of treatment depends on the size, location, and stage of the cyst. Surgical approaches range from cystectomy to nephrectomy in more severe cases. Several case studies and literature reviews have been published to provide insights into the presentation, diagnosis, and management of renal hydatid cysts. These studies highlight the importance of early detection and appropriate treatment to prevent complications and improve patient outcomes. While renal hydatid cyst is a rare manifestation of hydatid disease, it is essential for healthcare professionals specially urologists and pathologists to be aware of its potential occurrence, particularly in endemic regions. Early diagnosis and a multidisciplinary approach to treatment, combining pharmacotherapy and surgical interventions, are crucial for the successful management of this condition [1,2].

We present the case of a 59-year-old female who experienced gross hematuria and flank pain for one month. Initial radiologic assessments suggested malignancy. However, following a radical nephrectomy, the pathological evaluation confirmed the presence of a renal hydatid cyst, shifting the diagnosis from cancer to this rare parasitic infection.

Although the initial diagnosis was renal malignancy based on radiologic findings, alternative differential diagnoses, such as hydatid cyst, were not thoroughly considered prior to surgery. This oversight highlights the importance of integrating clinical, radiologic, and serologic evaluations to rule out hydatid disease, especially in endemic areas.

## 2 Case presentation

**Fig. 1:**
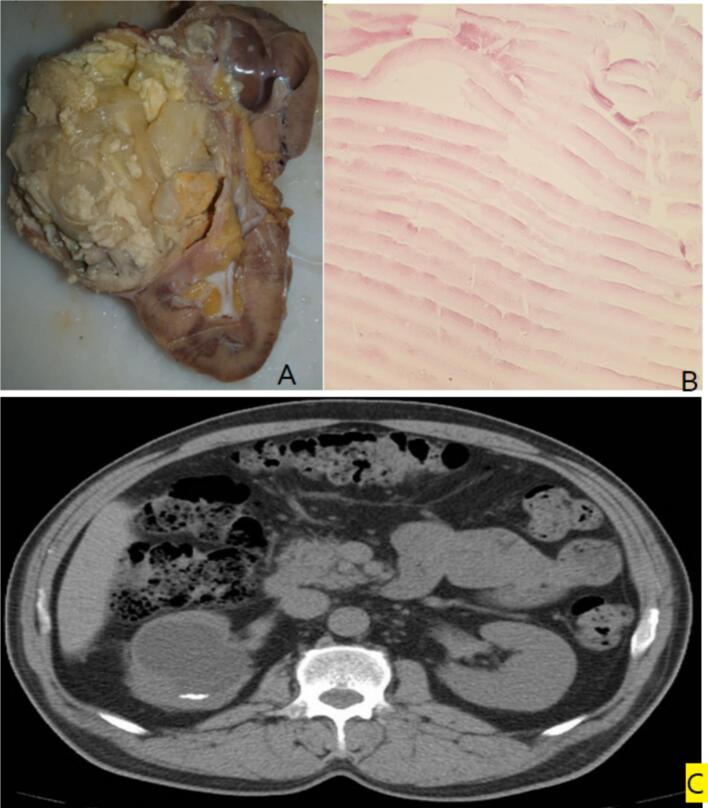
A: Macroscopic features of renal mass B: Microscopic features of renal mass C: CT scan evaluation of renal mass.

A 59-year-old woman presented to the hospital with a one-month history of right flank pain and gross hematuria. During the physical examination, her vital signs were stable, and the systemic examination revealed no significant abnormalities aside from mild dull pain in the right flank. To investigate her symptoms, an abdominal and pelvic ultrasound was performed, which identified a well-defined cystic lesion in the right kidney, featuring peripheral calcifications. This finding was further confirmed by a contrast-enhanced CT scan, which showed a large, irregular cystic mass with calcifications (Fig. 1C). Based on these radiologic findings, a preliminary diagnosis of renal malignancy was made.

Laboratory tests, including renal and liver function assessments, were normal. Urinalysis showed the presence of blood and numerous red blood cells, giving the urine a bloody appearance. Tumor markers, such as carcinoembryonic antigen (CEA) and alpha-fetoprotein (AFP), were within normal ranges. Given the size of the lesion and the suspected diagnosis, the patient was prepared for surgery, and a radical nephrectomy was performed.

Pathological examination of the excised kidney revealed a well-defined irregular cystic area measuring 7x7x6 cm, filled with whitish material (Fig. 1A). Multiple sections were taken for microscopic evaluation, which revealed a thick fibrous capsule made of connective tissue and an acellular, laminated, eosinophilic layer consistent with a laminated membrane (Fig. 1B). These histologic findings confirmed the diagnosis of a renal hydatid cyst.

The patient had an uneventful recovery following surgery and was discharged three days later. Upon receiving the pathological diagnosis, the surgeon recommended a three-month course of albendazole to prevent recurrence. At a follow-up appointment six months later, the patient remained asymptomatic with normal renal function and no evidence of hydatid cysts in other organs. The work has been reported in line with the SCARE criteria [18].

The patient did not receive preoperative albendazole therapy, which is commonly recommended to prevent cyst dissemination during surgery. In this case, the decision to proceed without preoperative medical therapy was due to the mistaken diagnosis of malignancy. This underscores the importance of considering hydatid cysts in the differential diagnosis before deciding on a surgical approach.

The decision to perform a radical nephrectomy was driven by the presumption of renal malignancy. Alternative surgical options, such as cystectomy or conservative surgery, were not considered due to the initial misdiagnosis. Had hydatid disease been suspected, a less aggressive approach aimed at cyst removal and kidney preservation could have been attempted.

## 3 Discussion

As hydatid disease usually involves the liver and lungs, kidneys are a very special target. Around 2 %–4 % of total cases are characterized by renal hydatid cysts [3]. Renal hydatid is a disease that can be hard to diagnose and very uncommon in comparison with liver or lung hydatid cysts, due to the absence of specific symptoms or indicators [4]. For many years, renal hydatid cysts may remain asymptomatic and thereafter can be found incidentally [1]. The symptoms and signs of hydatid cyst disease are dependent on the affected organ, the site and the secondary spread. The most common clinical findings are palpable masses [5,6]. In approximately 18 to 20 % of cases, rupture of a cyst into the urinary tract may result in hydatid urea and serve as an indicator for renal hydatidosis. In some patients, symptoms such as lower back pain, hematuria or abdominal lump may occur [2,7]. The main approach for treatment is surgery, as other methods are not highly successful. The treatment choices for renal hydatid cysts vary from simple cyst removal to a more aggressive nephrectomy [8]. The surgical method chosen depends on various factors such as the number of cysts in or outside the kidney, the extent of surrounding structure involvement, cyst size, and the outcomes of renal function tests. Both laparotomy and laparoscopic methods are used. In situations where only the kidneys are affected, laparoscopic surgery is increasingly favored because of its enhanced safety and ability to yield satisfactory outcomes [9]. When the goal is to preserve the renal tissue or when the urinary collecting system is not involved, percutaneous drainage may be employed as an alternative to surgery [10]. The significant potential for disease spread exists in these conditions [9]. As prophylaxis, albendazole is administered orally at 10–15 mg/kg/day (maximum400 mg) before and after surgery [1].

The reported cases of renal hydatid cysts over the past 20 years are summarized in Table 1. Among these cases, only one was treated exclusively with medical therapy, while the rest required surgical intervention. Nephrectomy was performed in three cases, including the present case, while cystectomy was performed in the others. Laparoscopic surgery was used in 10 of the cases.

**Table 1:** The reported cases of renal hydatid cysts over the past 20 years.

Author	Year	Country	Case	Sign Symptom	Treatment
Bilen et al. [11]	2006	Turkey	13 y/o male	Abdominal pain	Laparoscopic cystectomy
Basiri et al. [12]	2006	Iran	73 y/o male	Weight loss, foul smelling urine	Laparoscopic partial nephrectomy
Rabii et al. [13]	2006	Morocco	26 y/o female	Abdominal pain	Laparoscopic cystectomy
Shah et al. [14]	2009	India	45 y/o female	Abdominal pain	Laparoscopic nephrectomy
Prabhudessai et al. [15]	2009	India	30 y/o female	Abdominal pain	Laparoscopic cystectomy
Divarci et al. [16]	2010	Turkey	17 y/o female	Abdominal pain	Laparoscopic cystectomy
Aggarwal et al. [9]	2014	India	40 y/o female	Abdominal pain	Laparoscopic cystectomy
Aggarwal et al. [9]	2014	India	40 y/o male	Abdominal lump	Laparoscopic cystectomy
Aggarwal et al. [9]	2014	India	60 y/o female	Abdominal lump, weight loss, anorexia	Laparoscopic cystectomy
Soares et al. [7]	2015	Portugal	14 y/o male	Low back pain, hematuria	Medical
Paramythiotis et al. [6]	2016	Greece	44 y/o female	Chronic epigastric pain, mild fever	Laparotomy cystectomy
Anskievski et al. [17]	2017	Bulgaria	21 y/o female	Flank pain	Laparotomy cystectomy
Hafezi et al. [2]	2019	Iran	26 y/o female	Abdominal pain, hematuria	Laparotomy cystectomy
Banset et al. [1]	2021	Nepal	24 y/o female	Flank pain, fever	Laparotomy cystectomy
Current study	2024	Iran	59 y/o female	Flank pain, hematuria	Laparotomy nephrectomy

In the current case, a laparotomic nephrectomy was performed due to an initial diagnosis of malignancy, without preoperative medical treatment. Following the pathological diagnosis of hydatid cyst, postoperative medical prophylaxis was initiated, and the patient underwent routine follow-up appointments. This case illustrates the successful management of a large renal hydatid cyst without significant complications, emphasizing the importance of early and correct detection and meticulous surgical intervention.

Long-term follow-up of 12–24 months is recommended to monitor for recurrence of hydatid disease. In this case, follow-up was limited to six months, which may not be sufficient to entirely exclude the possibility of recurrence.

## 4 Conclusion

In conclusion, this case highlights the successful management of a large renal hydatid cyst through timely surgical intervention, underscoring the critical role of early detection in preventing complications. The case also demonstrates the importance of considering hydatid disease in the differential diagnosis of renal cystic masses, especially in regions where the disease is endemic. A combination of thorough preoperative evaluation, appropriate surgical technique, and postoperative medical prophylaxis ensured an optimal outcome for the patient, with no recurrence or complications in follow-up. This reinforces the value of a multidisciplinary approach in the management of rare parasitic conditions like renal hydatid cysts.

## CRediT authorship contribution statement

Maryam Maghbool [conceptualization, methodology and data curation] Mohammadreza Dehghani [writing, original draft and editing] Babak Samizadeh [conceptualization, methodology and data curation and submission].

## Consent

Written informed consent was obtained from the patient for publication and any accompanying images. A copy of the written consent is available for review by the Editor-in-Chief of this journal on request.

## Ethical approval

This study was conducted in full compliance with ethical standards and received approval from the Ethics Committee of Fasa University of Medical Sciences under the ethical code IR.FUMS.REC.1403.109.

## Guarantor

Maryam Maghbool.

## Research registration number

Not applicable.

## Funding

The authors received no financial support for the research, authorship, and/or publication of this article.

## Declaration of competing interest

None.
